# Correlation between nutritional assessment and oxidative stress in candidates for liver transplant

**DOI:** 10.31744/einstein_journal/2020AO4039

**Published:** 2019-12-06

**Authors:** Ana Carolina Cavalcante Viana, Fernanda Maria Machado Maia, Natália Sales de Carvalho, Suelyne Rodrigues de Morais, Alane Nogueira Bezerra, Priscila da Silva Mendonça, Sâmia Lopes da Costa, Ana Filomena Camacho Santos Daltro

**Affiliations:** 1 Hospital Universitário Walter Cantídio, Universidade Federal do Ceará, Fortaleza, CE, Brazil.; 2 Universidade Estadual do Ceará, Fortaleza, CE, Brazil.; 3 Universidade de Fortaleza, Fortaleza, CE, Brazil.; 4 Centro Universitário Christus, Fortaleza, CE, Brazil.

**Keywords:** Nutrition assessment, Oxidative stress, Antioxidants, Eating, Liver cirrhosis

## Abstract

**Objective:**

To evaluate the oxidative profile, nutritional status and food intake (caloric value; macronutrients; vitamins A, E and C; and zinc), and to correlate oxidative stress with nutritional status in patients who were candidates for liver transplant.

**Methods:**

This is a cross-sectional, analytical, and descriptive study with 51 candidates for liver transplant. Sociodemographic and clinical data, anthropometric parameters, food consumption, and a 10mL blood sample were collected from each patient. Oxidative stress was analyzed by the thiobarbituric acid method. The consumption of macronutrients, caloric value and micronutrients (zinc, vitamins A, E and C) were qualitatively analyzed, and zinc was also quantitatively analyzed.

**Results:**

The mean age was 49.17±8.17 years. The highest percentage of malnutrition was according to arm muscle circumference (56.86%), followed by arm circumference (52.94%), triceps skin fold (50.98%), and body mass index (1.96%). The mean malondialdehyde level was 14.80±8.72μM/L, presenting a negative correlation with the body mass index for patients with liver cirrhosis according to IMC-Campillo values (p=0.001; r=-0.430). Low energy, carbohydrate, protein, vitamin A and E consumption were observed in more than 50% of subjects.

**Conclusion:**

This study showed an association of nutritional status through body mass index for patients with liver cirrhosis according to IMC-Campillo, with oxidative stress in patients with liver cirrhosis on a liver transplant waiting list.

## INTRODUCTION

The liver is an organ responsible for important functions in the body, such as gluconeogenesis, lipid and protein metabolism, bile formation, and plasma protein synthesis. The involvement of this organ can lead to severe metabolic disorders, raising the individual’s risk of morbidity and mortality.^1^

In some cases, when there are no other therapeutic possibilities for liver disease, the choice of management may be liver transplant.^2^

In 2017, there were 2,109 liver transplants in Brazil, 201 of them in Ceará, which ranks fourth among the states that perform more organ transplants.^3^

Nutrition is one of the treatment pillars for uncompensated liver disease and for those who await a transplant. Liver diseases manifest with great repercussion on the nutritional status due to hypercatabolism and hypermetabolism. The compromise of micronutrient, carbohydrate, protein, and lipid absorption also contributes towards development of malnutrition in these individuals.^4^

The energy source of cirrhotic patients is also modified, since 75% of energy reserve comes first from fat, which represents almost twice what occurs in healthy individuals.^5^ With this perspective, protein-energy malnutrition rapidly is installed, becoming a cycle: deficiency of antioxidants causing greater hepatocellular damage due to oxidative stress, worsening of the disease, and raising risk of the patient’s morbidity and mortality.^6^

The diagnosis of malnutrition is complex in these patients, considering there is no gold standard for the nutritional diagnosis of a liver disease; it is recommended to apply more than one type of nutritional evaluation.^7^ Nutritional deficiency leads to modification in the oxidative and antioxidant mechanisms, which, in imbalance, contribute mostly to liver necrosis.^8^

Oxidative imbalance in alcoholic hepatitis, one of the most common types of hepatites, is caused by some factors, such as increased production of free radicals from ethanol metabolism and damaged mitochondria; increased number of nicotinamide adenine dinucleotides, resulting from ethanol and acetaldehyde oxidation; and reduction of antioxidants, such as glutathione, carotenoids and vitamin E, due to low food consumption, which is common in alcoholics.^8^

Oxygen-free radicals are the primary causes of tissue damage. Their direct action on DNA aggravates the hepatocellular lesion and affords the appearance of hepatocarcinoma. Under these conditions, the cell membrane suffers the action of oxidative stress due to lipid peroxidation, producing reagent aldehydes, such as malondialdehyde (MDA). Analysis of MDA is studied at depth in order to obtain the oxidative profile of individuals in various health situations.^6^

Foods rich in antioxidants decrease liver and systemic inflammation. Thus, the consumption of antioxidant agents is important for the treatment and stability of the disease. Micronutrients, such as vitamins A, E and C, can influence the inhibition of lipid peroxidation caused by free radicals and promote protection of cell membranes.^9^

The number of patients in the waiting list for liver transplant is high and it is growing. The *Centro de Transplante Hepático do Ceará* [Liver Transplant Center of Ceará] is a reference in this modality and fosters research that seeks better care for this population. It is important to understand the nutritional factors related to the course of liver disease to deliver efficient care to those awaiting transplant.

## OBJECTIVE

To evaluate the oxidative profile, nutritional status, and food consumption, and to correlate oxidative stress with a nutritional parameter in candidates to liver transplants.

## METHODS

This is a cross-sectional, analytical and descriptive study, conducted from March 2015 to November 2016, with cirrhotic candidates for liver transplant. Data were collected at the Nutrition Outpatient Clinic of the Liver Transplant Center of Ceará, *Hospital Universitário Walter Cantídiso* (HUWC), *Universidade Federal do Ceará* (UFC). The study was approved by the Research Ethics Committee, under opinion no. 446.502, CAAE: 15503123.2.0000.5534.

The convenience sample comprised 51 liver disease patients, aged from 19 to 59 years, who were candidates for liver transplants and under care during the study period at the said outpatient clinic. Those who were receiving enteral nutrition through a feeding tube, were bedridden, and had amputations were excluded.

The participant read and signed the Informed Consent Form, and afterwards information was collected from the medical records on sociodemographic (sex, age, and level of schooling) and clinical (underlying disease, Model for End-Stage Liver Disease – MELD^1^ information, Child-Pugh score,^10^ presence of *diabetes mellitus* - DM - and/or hypertension − HTN).

Next, an anthropometric assessment was made directly with each patient, including measurements of current weight, dry weight, height, body mass index (BMI), arm circumference (AC), triceps skinfold (TSF), and arm muscle circumference (AMC). The latter was obtained by using the following calculation: AMC (cm) = Arm circumference (cm) – π × [TSF (mm)/10].^11^

The dry weight, proposed by James, was obtained by substracting edema and ascites.^12^ Scales with a stadiometer (Filizola^®^, Brazil) were used to check weight and height. Arm circumference was measured using a flexible, non-extensible anthropometric tape measure (Sanny^®^, Brazil) and for TSF, an adipometer was employed (Lange^®^). Nutritional diagnosis was based on the classification by Campillo et al.,^13^ and AC, TSF, and AMC were classified according to Blackburn et al.^11^

Arm circumference, AMC, and TSF data were classified into four categories (malnutrition, eutrophic, overweight and obesity), whereas the BMI-Campillo^13^ was classified into two categories, malnutrition and eutrophic, as proposed by the author.

The BMI classification as per Campillo et al.,^13^ validated for cirrhotic patients, was made with the following reference values: BMI lower than 22kg/m^2^ classified the patient with no ascites as malnourished; lower than 23kg/m^2^ classified malnutrition in patients with light to moderate ascites; and lower than 25kg/m^2^ classified malnourished patients with severe ascites; patients with levels higher than these values were classified as non-malnourished or eutrophic.^13^

Serum concentrations of oxidative stress markers were analyzed by means of collection of 10mL of blood in tubes without anticoagulants, via peripheral venous puncture, from all the participating subjects. This analysis was done using blood serum from the individuals according to the method described by Buege et al., with modifications. This method consists of the reaction of MDA with thiobarbituric acid, and its product is detected by spectrophotometric reading.^14^

The cut-off point used for MDA was 1.1μM/L in healthy individuals, considering values ≥1.1μM/L as high oxidative stress, and ≤1.1μM/L as low oxidative stress.^15^

To evaluate food consumption, a 24-hour food diary was used as a collection tool, which was applied on two alternating days of the week. The first collection was in person, and the other, by telephone call. The conversion of home measurements into grams in reference to food diaries was made as per Pinheiro et al.^16^

Calculation of the nutritional value and calories of foods consumed and recorded in the R24h was made by means of the Dietwin Plus^®^ software.

Zinc consumption was obtained quantitatively by simple mean of values, and was statistically analyzed with the Estimated Average Requirement (EAR).^17^ The consumption of vitamins A, E, and C, as well as of carbohydrates, lipids, and proteins, was qualitatively analyzed. Analysis of the mean food consumption of vitamins was done by direct comparison with the EAR, Recommended Dietary Allowance (RDA), and Tolerable Upper Intake Level. Classification was as follows: lower than the RDA was considered lower than the recommendation; adequate, when the patient presented with ingestion higher than the RDA; and above the recommendation, when the patient had ingestion higher than the Tolerable Upper Intake Level.^18^

Carbohydrates and lipids were assessed by direct comparison of the percentage of the mean ingestion with the percentage recommended for pre-liver transplant,^1^ which establishes 70 to 80% of total energy value for carbohydrates, and 20 to 30% for lipids.

The mean of protein consumption and the mean of total energy ingestion were evaluated by direct comparison with the recommendation of adequate ingestion before liver transplant of 1.2 to 1.5g/kg/day for protein, and of 35 to 40Kcal/kg/day for calorie consumption,^4^ bearing in mind the ideal weight with a mean BMI of 21kg/m^2^ for women and 22kg/m^2^ for men.^19^

Consumption of carbohydrates, protein, and lipids was classified as insufficient when the mean ingestion was lower than the recommended values; adequate when the mean ingestion was within the recommended value range; and excessive when the consumption was greater than recommended.

Statistical analysis was obtained by standard deviation (SD), simple arithmetic mean, and correlation for nonparametric data by means of Spearman’s test, performed using the (SPSS) software, version 22. Statistical significance was defined as p<0.05.

## RESULTS

The sample was composed of 51 individual candidates to liver transplants, mean age of 49.17±8.17 years. The characterization of the population studied is shown on table 1 .

**Table 1:** Sociodemographic and clinical variables of pre-liver transplant patients

Variables	Population	Mean±SD
	n (%)	
Age, years		49.17±8.17
Male sex	31 (60.78)	
Level of schooling		
None	2 (3.92)	
Elementary or Middle School	41(80.39)	
Higher education	8 (15.69)	
MELD		18.72±4.02
Child-Pugh*		
A	8 (15.69)	
B	29 (56.86)	
C	11 (21.57)	
Comorbidities (DM and/or HTN)	20 (39.22)	

* Three (5%) individuals did not present with established Child-Pugh. SD: standard deviation; MELD: Model for End-Stage Liver Disease; DM: *diabetes mellitus* ; HTN: hypertension.

The most commonly found diagnosis in the population of this research was cirrhosis due to viral hepatitis and due to alcohol ingestion, as per table 2 .

**Table 2:** Diagnoses of pre-liver transplant patients

Diagnosis	Individuals with respective diagnosis
Alcoholic cirrhosis	17 (33.33)
Cirrhosis due to viral hepatites	16 (31.37)
Cryptogenic cirrhosis	8 (15.69)
Cholestatic cirrhosis and/or autoimmune hepatitis	3 (5.88)
Budd-Chiari syndrome	3 (5.88)
Cirrhosis and neuroendocrine tumor	1 (1.96)
Non-alcoholic steatohepatitis	1 (1.96)
Niemann-Pick	1 (1.96)
Retransplant due to rejection and cirrhosis	1 (1.96)

Result expressed as n (%).

The classification of nutritional status, according to table 3 , was verified with the use of BMI, AC, TSF, and AMC as anthropometric parameters, which presented means of 25.90±5.14kg/m^2^, 28.59±5.50cm, 16.61±8.50mm, and 25.37±3.69cm, respectively. For AC, TSF, and AMC, data were classified as malnutrition, eutrophia, obesity; for BMI-Campillo, they were classified as malnutrition and eutrophia.

**Table 3:** Classification of the nutritional status of pre-liver transplant patients

Classification of nutritional status	BMI-Campillo	AC	TSF	AMC
Malnutrition	16 (31.37)	27 (52.94)	26 (50.98)	29 (56.86)
Eutrophia	35 (68.63)	17 (33.33)	5 (9.80)	22 (43.14)
Overweight		3 (5.88)	1 (1.96)	0 (0.00)
Obesity		4 (7.84)	19 (37.25)	0 (0.00)

Result expressed as n (%). BMI: body mass index; AC: arm circumference; TSF: triceps skinfold; AMC: arm muscle circumference.

Qualitative assessment of energy consumption, carbohydrate, protein, lipid, and vitamins A, E, C, as well as the quantitative and qualitative analyses of zinc, are displayed on table 4 . We highlight the consumption of protein, in which 56.86% of population presented with a lower than recommended consumption.

**Table 4:** Analysis of calorie consumption adequacy; carbohydrate; protein; lipid; vitamins A, E, and C; and zinc of pre-liver transplant patients, as per recommendation

Calories and nutrients	Consumption	Below	Adequate	Above
EC, kg/IW	31.00±11.41	34 (66.67)	8 (15.69)	9 (17.65)
Proteins, (g)/kg/IW	1.23±0.50	29 (56.86)	14 (27.45)	8 (15.69)
Carbohydrates, %	60.49±7.78	45 (88.24)	5 (9.80)	1 (1.96)
Lipids, %	24.47±6.34	12 (23.53)	30 (58.82)	9 (17.65)
Vitamin A, mcg	1,010.64± 2,042.66	39 (76.47)	8 (15.69)	4 (7.84)
Vitamin E, mg	5.21±3.43	51 (100.00)	0 (0.00)	0 (0.00)
Vitamin C, mg	409.66±672.24	11(21.57)	35 (68.63)	5 (9.80)
Zinc, mg	7.20±3.93			
Qualitative analysis		41 (80.39)	10 (19.61)	0 (0.00)
Quantitative analysis		18 (35.29)	33 (64.71)	

Result expressed as mean±standard deviation and n (%); EC: energy consumption; IW: ideal weight.

**Figure 1:**
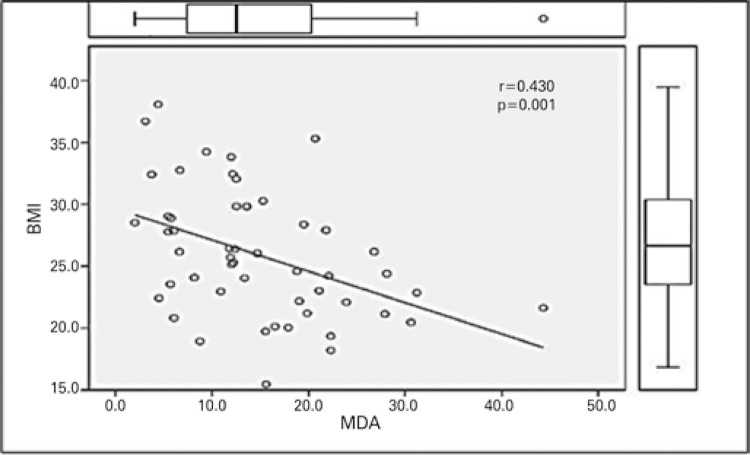
Correlation (Spearman’s test) of malondialdehyde and body mass index of pre-liver transplant patients BMI: body mass index; MDA: malondialdehyde.

The MDA mean was 14.80±8.72μM/L and all patients presented with values superior to reference for characterization of oxidative stress. The correlation of MDA with BMI-Campillo was shown to be significant (p=0.001; r=-0.430), as exposed in figure 1 .

## DISCUSSION

The population studied presented with alcoholic cirrhosis (33.33%) as the most prevalent diagnosis, followed by viral hepatites (31.37%), and differing from the study by Daltro et al.,^20^ in which alcoholic cirrhosis (24.32%) ranking second in prevalence.

The nutritional diagnosis in the patient with liver disease may be faulty due to the presence of fluid retention, which reduces sensitivity of the anthropometric datum. The BMI is liable to superestimation^1^ when the patient is swollen. With this perspective, higher cut-off points than those used by the World Health Organization have been suggested for cirrhotic patients.^13^

Using the values recommended by Campillo et al.,^13^ BMI presented with 31.37% malnutrition, which agrees with the high number of malnutrition cases indicated by other anthropometric parameters of the present study.

In another study, a higher percentage of malnourished individuals were identified, according to their TSF (49.0%) and AMC (28.6%),^7^ equivalent to what was found in the present study, which presented with a greater number of malnourished patients, according to AMC (56.86%) and TSF (50.98%). Some authors defend that the anthropometric data that uses upper limbs, such as TSF and AMC, are good indicators to confirm protein-energy malnutrition, since they suffer no interference from fluid retention.^7^

Malnutrition increases the risk of complications, such as hepatic encephalopathy, low level of immunity, elevated morbidity and mortality, and longer hospital stay.^21^

More than half of the studied presented with insufficient energy consumption, as well as of proteins and carbohydrates, corroborating what was found in the study by Ferreira et al., although these authors reported no ingestion de micronutrients.^22^ Adequate protein ingestion has an important effect on the gain of lean mass, in the synthesis of plasma proteins, and in the homeostasis balance of glutathione, which elevates the efficiency of the antioxidant system.^23^

The oxidative imbalance responsible for liver tissue damage in diseases that affect the liver can be attenuated with sufficient ingestion of antioxidant micronutrients.^24^ One antioxidant that has been studied at length is vitamin E, which stabilizes mismatched electrons, prevents lipid peroxidation, reduces the tumor necrosis factor in alcoholic hepatitis, and prevents the activation of stellate cells, which are responsible for hepatic fibrogenesis.^25^ In the present study, all individuals presented with insufficient consumption of this nutrient. The same was found in another study, in which 68.6% of population with nonalcoholic liver steatosis also had lower than recommended consumption.^26^

As to ascorbic acid, 68.63% of individuals presented with adequate consumption in this study, which does not differ from the investigation already mentioned in patients with nonalcoholic fatty liver disease, in which 72.8% presented with adequate ingestion.^26^ This vitamin has the capacity to directly neutralize the reactive oxygen species in the cell aqueous medium, which makes its consumption recommendable.^27^

The consumption of zinc was proved adequate in 64.71% of population evaluated in the present study. This result corroborated that of another study, according to which, 64% of cirrhotic patients presented with adequate ingestion of this nutrient.^28^

As provitamin A, beta-carotene works as a singlet oxygen and peroxyl radical carrier, acting as an antioxidant.^29^ The consumption of vitamin A was shown to be insufficient in 76.47% of this population studied, while, in general, less than half the individuals (42.4%) consumed it below the required level.^26^

Another finding in this study was the association between the value of BMI and oxidative stress, in that stress is presented at a higher level in the patient with a lower BMI. This can occur due to the clinical and nutritional status of the individual, and consequently, due to the reduction of antioxidant agents in the body and advanced liver disease.

According to the literature, concentrations of glutathione, primary cellular antioxidant, are significantly reduced in response to protein malnutrition, oxidative stress, liver diseases, and other conditions. In this way, reduction of glutathione contributes towards oxidative imbalance and the advance of liver disease, due to hepatocellular damage caused by free radicals.^30^

This study had a considerably elevated number of participants in comparison with the other studies cited here,^7 , 20 , 28^ which provides greater credibility to the data highlighted in this research. Additionally, its design does not seem to have been applied before with this same population.

Thus, the results shown here contributed towards knowledge of nutritional aspects and of the oxidative profile of liver disease patients who await liver transplants.

A few limitations can be mentioned, such as the fact of the study having been cross-sectional, since it was not possible to accompany the patient along the course of the disease, as well as their eating habits and nutritional status. The present study analyzed nutritional consumption by means of a 24-hour diary, a method that requires the evaluated subject to remember the quantity and type of food eaten on the previous day, a fact that could cause suppression of information or underestimation of the quantity consumed.

In order to obtain more plausible data on the influence of nutritional status on oxidative stress, longitudinal studies, and with other methods of measurements are suggested.

## CONCLUSION

This study showed the association of the nutritional status with oxidative stress using the body mass index for patients with liver cirrhosis (as per BMI-Campillo) who were on the waiting list for liver transplants. Adequate food consumption for maintenance or repletion of the nutritional status influences nutritional status, which can influence oxidative stress.

Food ingestion of nutrients within the recommended range is suggested in the literature and is associated with gain in the patient’s lean and fat mass, with improvement of the individual’s nutritional status, which, on the other hand, can be related to their oxidative profile.
