# Supplementary material for: Correlation between nutritional assessment and oxidative stress in candidates for liver transplant
Source: Einstein (Sao Paulo). 2019 Dec 6;18:eAO4039. doi: 10.31744/einstein_journal/2020AO4039 (PMC6905167; doi:10.31744/einstein_journal/2020AO4039)
Supplement: Translation

# Translation: Correlação entre avaliação nutricional e estresse oxidativo em candidatos ao transplante hepático

**Authors:** Ana Carolina Cavalcante Viana, Fernanda Maria Machado Maia, Natália Sales de Carvalho, Suelyne Rodrigues de Morais, Alane Nogueira Bezerra, Priscila da Silva Mendonça, Sâmia Lopes da Costa, Ana Filomena Camacho Santos Daltro

**Affiliations:** 1 Hospital Universitário Walter Cantídio, Universidade Federal do Ceará, Fortaleza, CE, Brasil.; 2 Universidade Estadual do Ceará, Fortaleza, CE, Brasil.; 3 Universidade de Fortaleza, Fortaleza, CE, Brasil.; 4 Centro Universitário Christus, Fortaleza, CE, Brasil.

**Keywords:** Avaliação nutricional, Estresse oxidativo, Antioxidantes, Ingestão de alimentos, Cirrose hepática

## Abstract

**Objetivo:**

Avaliar o perfil oxidativo, o estado nutricional e o consumo alimentar (valor calórico; macronutrientes; vitaminas A, E e C; e zinco), e correlacionar o estresse oxidativo com o estado nutricional em pacientes candidatos ao transplante hepático.

**Métodos:**

Trata-se de estudo transversal, analítico e descritivo com 51 pacientes candidatos ao transplante hepático. Coletaram-se dados sociodemográficos e clínicos, parâmetros antropométricos, consumo alimentar e amostra de 10mL de sangue de cada paciente. O estresse oxidativo foi analisado por método do ácido tiobarbitúrico. O consumo de macronutrientes, do valor calórico e dos micronutrientes (zinco, vitaminas A, E e C) foi analisado qualitativamente, e o zinco também foi analisado quantitativamente.

**Resultados:**

A média de idade foi de 49,17±8,17 anos. Maior percentual de desnutrição esteve de acordo com circunferência muscular do braço (56,86%), seguida de circunferência do braço (52,94%), dobra cutânea triciptal (50,98%) e índice de massa corporal (1,96%). A média do malondialdeído foi de 14,80±8,72µM/L, apresentando correlação negativa com os valores do índice de massa corporal para pacientes com cirrose hepática segundo IMC-Campillo (p=0,001; r=-0,430). Observou-se baixo consumo de energia, carboidrato, proteína, e vitaminas A e E em mais de 50% dos indivíduos.

**Conclusão:**

Este estudo mostrou associação do estado nutricional, por meio do índice de massa corporal para pacientes com cirrose hepática segundo IMC-Campillo, com o estresse oxidativo em paciente com cirrose hepática em lista de transplante de fígado.

## INTRODUÇÃO

O fígado é um órgão responsável por importantes funções no organismo, como gliconeogênese, metabolismo do lipídio e proteínas, formação da bile e síntese de proteínas do plasma. O acometimento desse órgão pode levar a sérios distúrbios metabólicos, elevando o risco de morbidade e mortalidade no indivíduo.^1^

Em alguns casos, quando não há mais outras possibilidades terapêuticas para doença hepática, a forma de tratamento pode ser o transplante hepático.^2^

No ano de 2017, foram realizados 2.109 transplantes de fígado no Brasil, e 201 deles foram no Ceará, estado que se encontra em quarto lugar entre os que mais transplantaram.^3^

A alimentação é um dos pilares para o tratamento da doença hepática descompensada e para quem aguarda o transplante. A hepatopatia manifesta-se com grande repercussão no estado nutricional, devido ao hipercatabolismo e ao hipermetabolismo. O comprometimento da absorção de micronutrientes, carboidratos, proteína e lipídio contribui também para a instalação da desnutrição nesses indivíduos.^4^

A fonte energética dos pacientes cirróticos também é alterada, pois 75% da reserva energética é precocemente provinda da gordura, o que representa quase o dobro do que ocorre em indivíduos saudáveis.^5^ Nessa perspectiva, a desnutrição energético-proteica instala-se rapidamente, tornando-se um ciclo: deficiência de antioxidantes, propiciando maior lesão hepatocelular pelo estresse oxidativo, agravamento da doença e elevação do risco de morbimortalidade do doente.^6^

O diagnóstico de desnutrição é complexo nesses pacientes, tendo em vista que não existe padrão-ouro para diagnóstico nutricional para hepatopata, sendo recomendada a associação de mais de um tipo de método de avaliação nutricional.^7^ A deficiência nutricional leva a alterações nos mecanismos oxidantes e antioxidantes, que, em desequilíbrio, contribuem para grande parte da necrose hepática.^8^

O desequilíbrio oxidativo na hepatite alcóolica, uma das mais comuns hepatites, origina-se por alguns fatores: elevação da produção de radicais livres provenientes do metabolismo do etanol e pelas mitocôndrias lesadas; aumento do número de dinucleotídio de nicotinamida e adenina, decorrente da oxidação do etanol e do acetaldeído; e redução de antioxidantes, como glutationa, carotenoides e vitamina E, devido ao baixo consumo alimentar, comum em alcoolistas.^8^

Os radicais livres de oxigênio são as principais causas da lesão tecidual. Sua ação direta no DNA agrava a lesão hepatocelular e propicia o aparecimento de hepatocarcinoma. Nessas condições, a membrana celular sofre a ação do estresse oxidativo, devido à peroxidação lipídica, produzindo aldeídos reagentes, como o malondialdeído (MDA). A análise do MDA é muito estudada, para obter o perfil oxidativo de indivíduos em diversas situações de saúde.^6^

Alimentos ricos em antioxidantes diminuem a inflamação hepática e sistêmica. Assim, o consumo de agentes antioxidantes se faz importante para o tratamento e a estabilidade da doença. Micronutrientes, como vitaminas A, E e C, podem influenciar na inibição da peroxidação lipídica causada pelos radicais livres e promover a proteção das membranas celulares.^9^

O número de pacientes em fila de transplante hepático é elevado e crescente. O Centro de Transplante Hepático do Ceará, o qual é referência nessa modalidade, favorece pesquisas que visam ao melhor atendimento desse público. É necessário conhecer os fatores nutricionais relacionados ao curso da hepatopatia, a fim de promover assistência eficiente àqueles que aguardam o transplante.

## OBJETIVO

Avaliar perfil oxidativo, estado nutricional e consumo alimentar, e correlacionar o estresse oxidativo com parâmetro nutricional em candidatos ao transplante hepático.

## MÉTODOS

Trata-se de estudo transversal, analítico e descritivo, com pacientes portadores de cirrose candidatos ao transplante hepático no período de março de 2015 a novembro de 2016. O local de coleta foi o Ambulatório de Nutrição no Centro de Transplante de Fígado do Ceará do Hospital Universitário Walter Cantídio (HUWC) da Universidade Federal do Ceará (UFC). O estudo foi aprovado pelo Comitê de Ética e Pesquisa, sob o parecer 446.502, CAAE: 15503123.2.0000.5534.

A amostra foi constituída por conveniência por 51 pacientes hepatopatas candidatos ao transplante hepático e em atendimento no período de estudo no referido ambulatório. Incluíram-se na pesquisa pacientes listados para transplante hepático com idade de 19 a 59 anos. Foram excluídos aqueles em nutrição por sonda enteral, acamados e com amputação.

Após o participante ler e assinar o Termo de Consentimento Livre e Esclarecido, foram coletadas, do prontuário, informações sociodemográficas (sexo, idade e escolaridade), clínicas (doença de base, *Model for End-Stage Liver Disease* – MELD −,^1^ escore Child-Pugh,^10^ presença de *diabetes mellitus* – DM − e/ou hipertensão arterial sistêmica − HAS).

Em seguida, realizou-se avaliação antropométrica diretamente com cada paciente, composta pelas aferições de peso atual, peso seco, altura, índice de massa corporal (IMC), circunferência do braço (CB), dobra cutânea triciptal (DCT) e circunferência muscular do braço (CMB). Esse último foi obtido por intermédio do seguinte cálculo: CMB (cm) = CB (cm) – π × [DCT (mm)/10].^11^

O peso seco foi obtido do desconto de edema e ascite proposto por James.^12^ Para aferição do peso e da altura, utilizou-se balança com estadiômetro (Filizola^®^, Brasil). A CB foi obtida com o auxílio de fita antropométrica flexível, não extensível (Sanny^®^, Brasil) e, para a DCT, utilizou-se adipômetro da marca Lange^®^. A classificação do diagnóstico nutricional foi baseado na classificação de Campillo et al.,^13^ e em Blackburn et al., para classificação da CB, DCT e CMB.^11^

Os dados de CB, CMB e DCT foram classificados em quatro categorias (desnutrição, eutrofia, sobrepeso e obesidade), ao passo que o IMC-Campillo^13^ foi classificado em apenas duas categorias (desnutrição e eutrofia), como proposto pelo autor.

A classificação do IMC conforme Campillo et al.,^13^ validado para pacientes cirróticos, realizou-se com os seguintes valores de referência: IMC abaixo de 22kg/m^2^classificou paciente sem ascite como desnutrido; abaixo de 23kg/m^2^classificou desnutrição em pacientes com ascite de grau leve a moderado; e abaixo de 25kg/m^2^classificou como desnutrido pacientes com ascite severa; acima desses valores, a classificação era não desnutrido ou eutrófico.^13^

As concentrações séricas de marcadores do estresse oxidativo foram analisadas por meio da coleta de 10mL de sangue em tubos sem anticoagulante, por punção venosa periférica, de todos os sujeitos participantes. Essa análise foi realizada utilizando o soro do sangue do indivíduo, pelo método descrito por Buege et al., com modificações. Esse método consiste na reação do MDA com o ácido tiobarbitúrico, em relação ao qual seu produto é detectado pela leitura espectrofotométrica.^14^

O ponto de corte empregado para MDA foi de 1,1μM/L em indivíduos saudáveis, considerando valores ≥1,1μM/L como de elevado estresse oxidativo, e ≤1,1μM/L como baixo estresse oxidativo valores.^15^

Para avaliação do consumo alimentar, utilizou-se o recordatório alimentar de 24 horas como ferramenta de coleta, o qual foi aplicado em 2 dias alternados da semana. A primeira coleta foi presencial e, a outra, por telefone. A conversão das medidas caseiras em gramatura referente aos recordatórios alimentares foi realizada conforme Pinheiro et al.^16^

O cálculo do valor nutritivo e das calorias dos alimentos consumidos e registrados no R24h foi alcançado pelo software Dietwin Plus^®^.

O consumo de zinco foi obtido quantitativamente pela média simples dos valores e analisado estatisticamente com *Estimated Average Requirement* (EAR).^17^ O consumo das vitaminas A, E e C, assim como de carboidrato, lipídio e proteína, foi analisado de forma qualitativa. A análise da média do consumo alimentar das vitaminas se deu por comparação direta com a EAR, *Recommended Dietary Allowance* (RDA) e *Tolerable Upper Intake Level.* A classificação foi a seguinte: menor do que a RDA, considerou-se abaixo da recomendação; adequado, quando o paciente apresentou ingestão acima da RDA; e acima da recomendação quando o paciente apresentou ingestão acima da *Tolerable Upper Intake Level.* ^18^

O carboidrato e o lipídio foram avaliados pela comparação direta do percentual da média de sua ingestão com o percentual recomendado para pré-transplante hepático,^1^ o qual preconiza 70 a 80% do valor energético total para carboidrato e 20 a 30% para lipídio para pré-transplante hepático.

A média do consumo de proteína e a média da ingestão energética total foram avaliadas por comparação direta com a recomendação de ingestão adequada para pré-transplante hepático de 1,2 a 1,5g/kg/dia para proteína, e de 35 a 40Kcal/kg/dia para consumo calórico,^4^ tendo em vista o peso ideal com o IMC médio de 21 kg/m^2^para mulheres e 22kg/m^2^para homens.^19^

O consumo de carboidrato, proteína e lipídio foi classificado como insuficiente quando a média da ingestão foi menor que os valores preconizados; adequado quando a média da ingestão apresentou-se dentro das recomendações; e excessivo quando o consumo foi maior do que o recomendado.

A análise estatística foi obtida por desvio padrão (DP), média aritmética simples e correlação para dados não paramétricos por meio do teste de Spearman, realizado utilizando o software (SPSS), versão 22. O valor de p<0,05 foi definido como estatisticamente significante.

## RESULTADOS

A amostra foi composta por 51 indivíduos candidatos ao transplante hepático, com média de idade de 49,17±8,17 anos. A caracterização da população estudada encontra-se na tabela 1 .

**Tabela 1:** Variáveis sociodemográficas e clínicas de pacientes pré-transplante hepático

Variáveis	População	Média±DP
	n (%)	
Idade, anos		49,17±8,17
Sexo masculino	31 (60,78)	
Escolaridade		
Nenhuma	2 (3,92)	
Ensino Fundamental ou Médio	41(80,39)	
Ensino Superior	8 (15,69)	
MELD		18,72±4,02
Child-Pugh*		
A	8 (15,69)	
B	29 (56,86)	
C	11 (21,57)	
Comorbidades (DM e/ou HAS)	20 (39,22)	

* Três (5%) indivíduos não apresentavam Child-Pugh estabelecido. DP: desvio padrão; MELD: *Model for End-Stage Liver Disease* ; DM: *diabetes mellitus* ; HAS: hipertensão arterial sistêmica.

O diagnóstico mais encontrado na população desta pesquisa foi cirrose por hepatites virais e por ingestão de álcool, conforme tabela 2 .

**Tabela 2:** Diagnósticos de pacientes pré-transplante hepático

Diagnóstico	Indivíduos com respectivo diagnóstico
Cirrose alcóolica	17 (33,33)
Cirrose por hepatites virais	16 (31,37)
Cirrose criptogênica	8 (15,69)
Cirrose colestática e/ou hepatite autoimune	3 (5,88)
Síndrome de Budd-Chiari	3 (5,88)
Cirrose e tumor neuroendócrino	1 (1,96)
Esteato hepatite não alcóolica	1 (1,96)
Niemann-Pick	1 (1,96)
Retransplante por rejeição e cirrose	1 (1,96)

Resultado expresso por n (%).

A classificação do estado nutricional, conforme tabela 3 , foi verificada utilizando IMC, CB, DCT e CMB como parâmetros antropométricos, os quais apresentaram média de 25,90±5,14kg/m^2^, 28,59±5,50cm, 16,61±8,50mm e 25,37±3,69cm, respectivamente. Para CB, DCT e CMB, os dados foram classificados em desnutrição, eutrofia, obesidade; para IMC-Campillo, foram classificados em desnutrição e eutrofia.

**Tabela 3:** Classificação do estado nutricional de pacientes pré-transplante hepático

Classificação do estado nutricional	IMC-Campillo	CB	DCT	CMB
Desnutrição	16 (31,37)	27 (52,94)	26 (50,98)	29 (56,86)
Eutrofia	35 (68,63)	17 (33,33)	5 (9,80)	22 (43,14)
Sobrepeso		3 (5,88)	1 (1,96)	0 (0,00)
Obesidade		4 (7,84)	19 (37,25)	0 (0,00)

Resultado expresso por n (%). IMC: índice de massa corporal; CB: circunfência do braço; DCT: dobra cutânea triciptal; CMB: circunferência muscular do braço.

A avaliação qualitativa do consumo energético, de carboidrato, proteína, lipídio, e vitaminas A, E, C, assim como a análise quantitativa e qualitativa de zinco, estão expostas na tabela 4 . Destaca-se o consumo de proteína, em que 56,86% da população apresentou consumo abaixo da recomendação.

**Tabela 4:** Análise da adequação do consumo calórico; carboidrato; proteína; lipídio; vitaminas A, E e C; e zinco de pacientes pré-transplante hepático, conforme recomendação

Caloria e nutrientes	Consumo	Abaixo	Adequado	Acima
CE, kg/PI	31,00±11,41	34 (66,67)	8 (15,69)	9 (17,65)
Proteína, (g)/kg/PI	1,23±0,50	29 (56,86)	14 (27,45)	8 (15,69)
Carboidrato, %	60,49±7,78	45 (88,24)	5 (9,80)	1 (1,96)
Lipídio, %	24,47±6,34	12 (23,53)	30 (58,82)	9 (17,65)
Vitamina A, mcg	1.010,64±2.042,66	39 (76,47)	8 (15,69)	4 (7,84)
Vitamina E, mg	5,21±3,43	51 (100,00)	0 (0,00)	0 (0,00)
Vitamina C, mg	409,66±672,24	11(21,57)	35 (68,63)	5 (9,80)
Zinco, mg	7,20±3,93			
Análise qualitativa		41 (80,39)	10 (19,61)	0 (0,00)
Análise quantitativa		18 (35,29)	33 (64,71)	

Resultado expresso por média±desvio padrão e n (%); CE: consumo energético; PI: peso ideal.

A média de MDA foi de 14,80±8,72 μM/L e todos os pacientes apresentaram valores superiores aos de referência para caracterização de estresse oxidativo. A correlação do MDA com IMC-Campillo mostrou-se significativa (p=0,001; r=-0,430), conforme figura 1 .

**Figura 1:** Correlação (teste de Spearman) do malondialdeído e índice de massa corporal dos pacientes pré-transplante hepático IMC: índice de massa corporal; MDA: malondialdeído.

## DISCUSSÃO

A população estudada apresentou cirrose por álcool (33,33%) como diagnóstico de maior prevalência, seguido por hepatites virais (31,37%), diferindo da pesquisa de Daltro et al.,^20^ na qual a cirrose alcóolica (24,32%) foi a segunda causa de maior prevalência.

O diagnóstico nutricional no portador de doença hepática pode ser falho devido à presença de retenção de fluidos, o que reduz a sensibilidade do dado antropométrico. O IMC está passível à superestimação^1^ quando se trata de paciente edemaciado. Nessa perspectiva, pontos de corte maiores do que os usados pela Organização Mundial da Saúde foram sugeridos para pacientes cirróticos.^13^

Usando os valores recomendados por Campillo et al.,^13^ o IMC apresentou 31,37% de desnutrição, o que concorda com o elevado número de desnutrição apontado por outros parâmetros antropométricos do presente estudo.

Em outra pesquisa, foi identificado maior percentual de desnutridos, de acordo com a DCT (49,0%) e a CMB (28,6%),^7^ equivalente ao encontrado na presente pesquisa, a qual apresentou maior número de pacientes desnutridos, de acordo com a CMB (56,86%) e DCT (50,98%). Alguns autores defendem que os dados antropométricos que utilizam membros superiores, como DCT e CMB, são bons indicadores para evidenciar desnutrição proteico-energética, por não sofrerem interferência da retenção hídrica.^7^

A má nutrição aumenta o risco de complicações como encefalopatia hepática, baixa imunidade, elevação da morbidade e da mortalidade, e maior tempo de internação.^21^

Mais da metade da população estudada apresentou-se com consumo insuficiente de energia, assim como de proteínas e carboidratos, corroborando com o encontrado no estudo de Ferreira et al., entretanto esses autores não relataram a ingestão de micronutrientes.^22^ Ingestão adequada de proteína tem efeito importante no ganho de massa magra, na síntese de proteínas plasmáticas e no equilíbrio da homeostase da glutationa, o que eleva a eficiência do sistema antioxidante.^23^

O desequilíbrio oxidativo responsável pela lesão do tecido hepático em doenças que acometem o fígado pode ser atenuado com ingestão suficiente de micronutrientes antioxidantes.^24^ Um antioxidante bastante estudado é a vitamina E, que estabiliza elétrons desemparelhados, previne a peroxidação lipídica, reduz o fator de necrose tumoral na hepatite alcóolica, e previne a ativação das células estreladas, as quais são responsáveis pela fibrogênese hepática.^25^ No presente estudo, todos os indivíduos apresentaram consumo insuficiente desse nutriente. O mesmo foi encontrado em outro trabalho, no qual 68,6% da população com esteatose hepática não alcoólica também obteve consumo abaixo do recomendado.^26^

Em relação ao ácido ascórbico, 68,63% dos indivíduos apresentaram consumo adequado neste estudo, o que não difere da pesquisa já citada de pacientes com doença hepática gordurosa não alcoólica, na qual 72,8% apresentaram ingestão adequada.^26^ Essa vitamina tem a capacidade de neutralizar diretamente as espécies reativas de oxigênio no meio aquoso da célula, o que torna seu consumo recomendável.^27^

O consumo de zinco apresentou-se adequado em 64,71% da população avaliada no presente estudo. Esse resultado corroborou o de outra pesquisa, segundo a qual 64% dos pacientes cirróticos apresentaram ingestão adequada desse nutriente.^28^

Tratando-se da pró-vitamina A, o betacaroteno funciona como carregador de oxigênio *singlet* e de radicais peroxil, atuando como antioxidante.^29^ O consumo da vitamina A apresentou-se insuficiente em 76,47% desta população estudada, enquanto, em geral, menos da metade dos indivíduos (42,4%) o consume abaixo das necessidades.^26^

Outro achado neste estudo foi a associação entre o valor de IMC e o estresse oxidativo, de forma que o estresse se apresenta mais elevado no paciente com menor IMC. Isso pode ocorrer devido ao estado clínico e nutricional do indivíduo e, consequentemente, à redução de agentes antioxidantes no organismo e na doença hepática avançada.

De acordo com a literatura, concentrações de glutationa, principal antioxidante celular, são reduzidos significativamente em resposta à desnutrição proteica, ao estresse oxidativo, a hepatopatias e a outras condições. Dessa forma, a redução da glutationa contribui para desequilíbrio oxidativo e o avanço da doença hepática, devido à lesão hepatocelular promovida pelos radicais livres.^30^

Este estudo teve número consideravelmente elevado de participantes em comparação com os outros estudos aqui citados,^7 , 20 , 28^ o que fornece maior credibilidade aos dados apontados por esta pesquisa. Além disso, seu desenho parece não ter sido realizado antes com esse mesmo público.

Dessa forma, os resultados aqui apontados contribuem para o conhecimento de aspectos nutricionais e do perfil oxidativo de pacientes hepatopatas que aguardam o transplante de fígado.

Algumas limitações podem ser apontadas, como o fato de o estudo ter sido transversal, pois não foi possível acompanhar o paciente na evolução da doença, assim como seus hábitos alimentares e estado nutricional. O presente estudo analisou o consumo alimentar por recordatório de 24 horas, cujo método requer do avaliado a lembrança da quantidade e do alimento consumido no dia anterior, fato que pode acarretar supressão de informações ou subestimação da quantidade consumida.

Para se obterem dados mais plausíveis quanto à influência do estado nutricional no estresse oxidativo, sugerem-se estudos longitudinais e com outros métodos de mensuração do estresse.

## CONCLUSÃO

Este estudo mostrou associação do estado nutricional, por meio do índice de massa corporal para pacientes com cirrose hepática segundo IMC-Campillo, com o estresse oxidativo em paciente com cirrose hepática em lista de transplante de fígado. O consumo alimentar adequado para a manutenção ou a repleção do estado nutricional influencia no estado nutricional, que, por sua vez, pode influenciar no estresse oxidativo.

A ingestão alimentar de nutrientes dentro do recomendado é sugerida na literatura e se associa com ganho de massa magra e gorda do paciente, e com a melhora do estado nutricional do indivíduo, que, por sua vez, pode estar relacionado ao seu perfil oxidativo.
